# Magnitude and attributed reasons for adult weight gain amongst women at increased risk of breast cancer

**DOI:** 10.1186/s12905-022-02037-w

**Published:** 2022-11-12

**Authors:** Mary Pegington, Elaine F. Harkness, Anthony Howell, D. Gareth Evans, Michelle Harvie

**Affiliations:** 1 Division of Cancer Sciences, The University of Manchester, Wilmslow Road, Manchester, M20 4BX England; 2 The Prevent Breast Cancer Research Unit, The Nightingale Centre, Manchester University NHS Foundation Trust, Manchester, M23 9LT England; 3 Division of Informatics, Imaging and Data Science, Faculty of Biology, Medicine and Health, University of Manchester, Manchester Academic Health Science Centre, Manchester, M13 9PL England; 4 Manchester Breast Centre, Manchester Cancer Research Centre, University of Manchester, 555 Wilmslow Rd, Manchester, M20 4GJ England; 5 Genomic Medicine, Division of Evolution and Genomic Sciences, The University of Manchester, St Mary’s Hospital, Manchester University NHS Foundation Trust, Oxford Road, Manchester, M13 9WL England; 6 NW Genomic Laboratory Hub, Manchester Centre for Genomic Medicine, Manchester University Hospitals NHS Foundation Trust, Manchester, M13 9WL England; 7 Faculty of Biology, Division of Evolution and Genomic Sciences, School of Biological Sciences, Medicine and Health, University of Manchester, Manchester Academic Health Science Centre, Manchester, M13 9PL England

**Keywords:** Breast cancer, Weight gain, BMI, Women

## Abstract

**Background:**

Excess weight (BMI ≥25.0 kg/m^2^) and weight gain during adult life increase the risk of postmenopausal breast cancer in women who are already at increased risk of the disease. Reasons for weight gain in this population can inform strategies for weight gain prevention.

**Methods:**

Baseline data from six weight loss studies for women at increased risk of breast cancer (age 31–74 years) were collated. Self-reported patterns of adult weight gain and attributed reasons for weight gain before joining the weight loss study were reported for the whole population and secondary analyses reported the different reasons given by women with/without children, pre−/peri- or postmenopausal, and moderate/high risk of breast cancer.

**Results:**

Five hundred and one women with a mean age of 47.6 (SD 8.4) years and median BMI of 29.9 (IQR 27.0–34.7) kg/m^2^ were included in the analyses. The median weight gain since young adulthood (18–20 years) was 20.5 (IQR 14.0–29.7) kg or 33.7 (23.4–50.2) % and median annual weight gain was 0.73 (IQR 0.51–1.08) kg. Four hundred and one women were included in analysis of weight gain reasons. The main five self-reported reasons for weight gain were children / childcare / pregnancy (stated by 55.9% of participants), followed by inactivity (41.9%), comfort or boredom eating (38.2%), portion size (32.4%), and stress (27.4%). Reasons appeared broadly similar between the different groups in the secondary analyses.

**Conclusions:**

We have highlighted common reasons for weight gain in women at increased risk of breast cancer. This will inform future interventions to support women to avoid weight gain in adulthood which would reduce the burden of breast cancer.

**Trial registration:**

NIHR NRR N0226132725, ISRCTN52913838, ISRCTN77916487, ISRCTN91372184, ISRCTN10803394 and ISRCTN16431108.

## Background

Excess weight (BMI ≥25.0 kg/m^2^) [1] and weight gain during adult life increases the risk of postmenopausal breast cancer [2, 3]. Currently in England, 60% of adult women are overweight including 29% with obesity [4]. Breast cancer is the most common cancer in women worldwide [5], and the second highest cause of cancer death in UK women [6]. It is estimated that 8% of UK breast cancers are caused by overweight and obesity [7], equating to around 4500 cases per year [6]. Various groups have highlighted young adulthood (18–35 years) as the main time for weight gain in women (summarised in [8] and recent additional data from a large English cohort [9]). In addition to increasing risk of breast cancer, weight gain during adulthood increases risk of diabetes, cardiovascular disease and other cancers in females [10].

A significant proportion of breast cancer cases, around 40%, are reported to occur in the 20% of women who are at increased risk (lifetime risk of breast cancer ≥17% [11] or 1 in 6 as defined by the Tyrer Cuzick model [12]). Targeting higher risk women with health behaviour interventions will have the biggest impact on reducing breast cancer rates. Observational studies have reported that the relative risks of higher BMI and adult weight gain apply equally to women already at increased risk of the disease, i.e. with a family history or high polygenic risk score, compared to lower risk women [13–15]. An Australian cohort study found that being overweight and obese is associated with a greater absolute increase in breast cancer risk amongst women already at high familial risk compared with women from the general population. They concluded that “maintaining a healthy weight throughout adult life is of clinical significance for all women, and especially those with a family history of breast cancer” [16].

Many women known to be at increased risk of breast cancer attend Family History, Risk and Prevention Clinics (FHRPCs) of which there are around 90 in the UK, including our own based at The Nightingale Centre, Manchester University NHS Foundation Trust (MFT). Analysis of BMI and health behaviour data from a sample of high risk women in our Manchester FHRPC (*n* = 136) highlighted the prevalence of unhealthy behaviours which were comparable to the general population, i.e. almost 60% had overweight or obesity, 30% did not meet physical activity (PA) recommendations, and 45% exceeded alcohol recommendations [17].

Since 2002 we have performed a number of weight loss intervention studies for women at increased risk of breast cancer with BMI ≥24 kg/m^2^ or weight gain > 10 kg since young adulthood. Findings of these studies have been published separately (Table 1). As part of the studies, baseline data was collected on 1) self-reported weight gain since the age of 18 or 20 years, and 2) women’s perceived reasons for this weight gain.

**Table 1:** Key information about the weight loss studies included in analyses

Study	Year of baseline weight	Age at self-reported young adult weight (y)	Key inclusion criteria				Ethics committee and registration number	Funding information	Trial registry details
			Breast cancer risk	Current weight	Adult weight gain	Menopausal status			
Lifestyle	2002	20	Risk 1 in 8 to 1 in 3	BMI < 40 kg/m^2^	Weight gain > 10 kg since age 18	Premenopausal	South Manchester REC (01/426)	Prevent Breast Cancer (registered charity number 1109839)	NIHR NRR N0226132725
IER vs CER diets	2009–10	18	Risk 1 in 6 to 1 in 3	BMI 24–45 kg/ m^2^ and / or body fat > 30%.	Weight gain > 7 kg since age 20	Any	Bolton (Lancashire) Local REC (05/Q1409/42)	Prevent Breast Cancer (as above) and Breast Cancer Now (registered charity number 1160558)	ISRCTN52913838
BRRIDE	2009–10	18	Risk ≥1 in 6	BMI 24–35 kg/ m^2^	Not defined	Premenopausal	North West 10 REC, Greater Manchester North (09/H1006/33)	Prevent Breast Cancer (as above)	ISRCTN77916487
PROCAS-Lifestyle	2014–16	18	Not defined	BMI ≥25 kg/m^2^	Not defined	Any	NRES Committee West Midlands–Solihull (14/WM/1088)	Prevent Breast Cancer (as above)	ISRCTN91372184
BRRIDE 2	2015	18	Not defined	BMI 30–45 kg/m^2^ plus body fat ≥40% or waist ≥88 cm. Weight < 125 kg	Not defined	Premenopausal	NRES Committee South Central - Oxford B (14/SC/1097)	Prevent Breast Cancer (as above)	ISRCTN10803394
Family History Lifestyle	2017–19	18	Moderately increased / high risk (> 17% lifetime risk)	BMI ≥25 kg/m^2^	Not defined	Any	North West - Preston Research Ethics Committee (17/NW/0440)	Prevent Breast Cancer (as above)	ISRCTN16431108

The aim of this paper is to report findings on the above two elements.

## Methods

### Data source

**Fig. 1:**
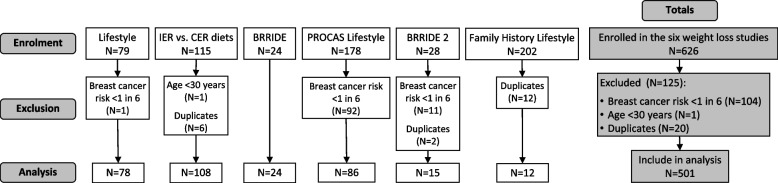
CONSORT 2010 flow diagram of the six included studies. IER = intermittent energy restriction, CER = continuous energy restriction, PROCAS = Predicting Risk of Cancer at Screening, BRRIDE = Breast Risk Reduction Intermittent Diet Evaluation

The analysis used anonymised baseline data from six weight loss studies conducted at the FHRPC between 2002 and 2019 (Fig. 1, *n* = 521) [18–23]. Inclusion criteria and details of ethics approvals and trial registry listings are given in Table 1.

### Measures

Weight and height at study entry were measured as per standard protocols. Weight at age 18 or 20 was recalled by the participant. BMI at age 18 or 20 and entry was categorised as underweight (< 18.5 kg/m^2^), healthy weight (18.5–24.9 kg/m^2^), overweight (25.0–29.9 kg/m^2^), and obese (≥30.0 kg/m^2^) [1]. Weight change since young adulthood (kg) is calculated as the difference between weight at joining the study, and young adulthood weight (18 or 20 years depending on study). Annual weight change was calculated as weight change since young adulthood (kg) / duration between young adulthood weight and study entry (years). Weight loss was defined as a loss of 5% or more between young adulthood and study entry [24], weight maintenance as maintaining between +/− 5% and the two weight gain categories were gaining 5–9.9%, or 10% or more. Participants reported their perceived opinions on reasons for weight gain using standard in-house questionnaires which included tick boxes for commonly occurring life events, psychological and physiological factors as well as actual diet and PA behaviours, with free text space for other reasons. Women could give more than one reason for weight gain. English Index of Multiple Deprivation was derived from postcodes [25].

### Data inclusion

We included women at increased risk of breast cancer (i.e. ≥17% lifetime risk) and age ≥ 30 years as we were interested in weight gain from young adulthood. Duplicates were removed as women could enter more than one study. Data from the first study that they entered was included in the analyses. Analysis of weight gain reasons included women who had both gained ≥5% weight and given reasons for their weight gain. Two of the included studies also recruited women from outside of the FHRPC, but the majority of women recruited attended the Manchester FHRPC.

### Statistical analysis

Descriptive statistics are presented with normally distributed data presented as mean (SD), otherwise median (IQR 25th–75th percentile) is used. Categorical data are presented as n (%). Reasons for weight gain are presented as a proportion of the included population stating each reason in descending order. Secondary analyses were run in sub-populations: those with / without children, pre / peri or postmenopausal at study entry, moderate risk of breast cancer (17–30% lifetime risk) / high risk of breast cancer (> 30% lifetime risk). Difference in reasons for weight gain between groups was compared using Pearson chi-square or Fisher’s exact tests. Bonferroni correction for multiple hypothesis testing was applied, by dividing the standard *p*-value by the number of comparisons made (0.05/30 = 0.0017). Analysis was performed using SPSS 25 (IBM, New York, USA).

## Results

Included data are shown in Fig. 1. Women were excluded who were at low or average risk of breast cancer (i.e. < 17% lifetime risk, *n* = 104) aged and age < 30 years (*n* = 1). Duplicates were removed as *n* = 20 women had been in more than study. Uptake to and withdrawal from the included studies is described in the published papers [19–21, 26]. Upon joining the weight loss studies, the population had a mean age of 47.6 (SD 8.4) years and a median lifetime breast cancer risk of 29.0 (IQR 22.4–33.3) %. They were predominantly of white ethnicity (96%) and from the two least deprived quintiles (55%, Table 2).

**Table 2:** Characteristics at young adulthood and joining weight loss study, and weight change between those times (*n* = 501)

	Young adulthood	At joining weight loss study
Age at joining weight loss study^a^	–	47.6 (8.4)
Height (m)^a^	–	1.64 (0.06)
Ethnicity^b^	–	
White		480 (95.8)
Asian or Asian British		8 (1.6)
Black or Black British		8 (1.6)
Mixed		2 (0.6)
Other		3 (0.4)
English Index of Multiple Deprivation quintile^b^	–	
1 (most deprived)		64 (12.8)
2		67 (13.4)
3		95 (19.0)
4		123 (24.6)
5 (least deprived)		152 (30.3)
Premenopausal / Peri or postmenopausal^b^	–	271 (54.7) / 224 (45.3) Missing *n* = 6
Parous^b^	–	392 (79.0) Missing *n* = 5
Lifetime breast cancer risk (%)^c^	–	29.0 (22.4–33.3) Missing *n* = 12
Weight (kg)^c^	60.3 (54.0–66.7)^d^ Missing *n* = 32	81.5 (72.8–93.5) Missing *n* = 0
BMI (kg/m^2^)^c^	22.1 (20.3–24.1)^d^ Missing n = 32	29.9 (27.0–34.7) Missing *n* = 0
BMI categories^b^:		
Underweight (< 18.5 kg/m^2^)	33 (7.0)	0 (0.0)
Healthy weight (18.5–24.9 kg/m^2^)	350 (74.6)	37 (7.4)
Overweight (25–29.9 kg/m^2^)	63 (13.4)	216 (43.1)
Obese (≥30.0 kg/m^2^)	23 (4.9)	248 (49.5)
Weight change since young adulthood (kg)^c^	–	20.5 (14.0–29.7) Missing *n* = 32
Weight change since young adulthood (%)^c^	–	33.7 (23.4–50.2) Missing *n* = 32
Duration between young adulthood weight and study entry (y)^a^	–	29.3 (8.6)
Weight change per year (kg)^c^	–	0.73 (0.51–1.08) Missing *n* = 32
Weight change category^b^:	–	
Lost ≤5%		6 (1.3)
Maintained (within ±5%)		6 (1.3)
Gained 5–9.9%		10 (2.1)
Gained ≥10%		447 (95.3) Missing *n* = 32

^a^mean (SD), ^b^n (%), ^c^median (IQR: 25th and 75th percentiles), ^d^young adulthood weight was self-reported

In early adulthood (age 18–20 years), 74.6% had a healthy range BMI whilst only 7.4% were in the healthy BMI range at study entry (age 31–74 years, all ≥24 kg/m^2^ as per eligibility criteria for the weight loss studies, Table 1). Only 2.7% (12/469) of the population either lost weight or maintained within ±5% of their young adulthood weight while 95.3% gained ≥10% weight. The median annual weight gain was 0.73 (0.51–1.08) kg (Table 2).

Amongst participants who had gained ≥5% weight since young adulthood and provided reasons for weight gain (*n* = 401), the median number of reasons given per participant was 4 (IQR 2–6). Two thirds of women gave three or more reasons for their weight gain. The main reason given for weight gain was children / childcare / pregnancy (stated by 55.9% of participants), followed by inactivity (41.9%), comfort or boredom eating (38.2%), portion size (32.4%), and stress (27.4%, Table 3).

**Table 3:** Self-reported reasons for weight gain for women with weight gain of ≥5% weight in adulthood (*n* = 401)

Reason		Number stating reason	% stating reason
1	Children / Childcare / Pregnancy	224	55.96^a^
2	Inactivity	168	41.9
3	Comfort or boredom eating	153	38.2
4	Portion Size	130	32.4
5	Stress	110	27.4
6	Marriage / settling down	96	23.9
7	Work / Study	81	20.2
8	Menopause / HRT	73	18.2^b^
9	Bereavement	73	18.2
10	Depression	64	16.0
11	Takeaways / Ready meals	61	15.2
12	Lack of time to prepare food	55	13.7
13	Alcohol	48	12.0
14	Food Preferences (own/family)	43	10.7
15	Giving up smoking	41	10.5
16	Health problems / Medications	38	9.5
17	Contraceptives	17	4.2
18	Cooking skills	11	2.7
19	Cost of healthy food	10	2.5
20	Eating out	6	1.5
21	Lack of sleep / tiredness	6	1.5
22	Carer	4	1.0
23	Fertility treatment	4	1.0
24	Social life / holidays	4	1.0
25	Sweet tooth	3	0.7
26	Divorce	2	0.5
27	Hysterectomy / oophorectomy	1	0.2
28	Appetite / Hunger	1	0.2
29	Premenstrual hunger	1	0.2
30	Progesterone replacement	1	0.2

Excluding women missing percentage weight change (*n* = 32), women with percentage weight gain ≤5% (*n* = 12), and women not giving reasons for weight gain (*n* = 72): women could have been excluded for more than one of these reasons. Total excluded = 100. Participants could give multiple reasons for weight gain

^a^Includes all women but only *n* = 327 women known to have children. Of those with children, 224/327 (68.5%) gave Children / Childcare / Pregnancy as a reason for their weight gain

^b^Includes all women but only *n* = 188 women known to be peri/postmenopausal women. Of peri/postmenopausal women, 73/188 (38.8%) gave menopause/HRT as a reason for their weight gain

None of the comparisons between the subgroups reached statistical significance after adjustment for multiple hypothesis testing (results not shown). For the sub-cohort with no children (*n* = 72), the most common reason for weight gain was inactivity (stated by 56.9%). Comfort or boredom eating, portion size, stress, and work / study as reasons for weight gain were all given by a higher proportion of women with no children compared to women with children (54.2% vs. 34.9, 41.7% vs. 30.6, 36.1% vs. 25.7, 27.8% vs. 18.7% respectively) (Table 4).

**Table 4:** Self-reported reasons for weight gain: women with and without children

Reason	Women with children (***n*** = 327)		Women without children (***n*** = 72)	
	Number stating reason	% stating reason	Number stating reason	% stating reason
Children / Childcare / Pregnancy	224	68.5	–	–
Inactivity	126	38.5	41	56.9
Comfort or boredom eating	114	34.9	39	54.2
Portion Size	100	30.6	30	41.7
Stress	84	25.7	26	36.1
Marriage / settling down	75	22.9	20	27.8
Work / Study	61	18.7	20	27.8
Menopause / HRT	58	17.7^a^	15	20.8^b^
Bereavement	59	18.0	14	19.4
Depression	48	14.7	16	22.2
Lack of time to prepare food	44	13.5	11	15.3
Takeaways / Ready meals	45	13.8	16	22.2
Alcohol	36	11.0	12	16.7
Food Preferences (own/family)	35	10.7	8	11.1
Giving up smoking	31	9.5	10	13.9
Health problems / Medications	33	10.1	5	6.9
Contraceptives	15	4.6	2	2.8
Cost of healthy food	10	3.1	0	0.0
Cooking skills	8	2.4	3	4.2
Lack of sleep / tiredness	6	1.8	0	0.0
Eating out	3	0.9	3	4.2
Fertility treatment	3	0.9	1	1.4
Social life / holidays	2	0.6	2	2.8
Sweet tooth	2	0.6	1	1.4
Carer	1	0.3	3	4.2
Divorce	1	0.3	1	1.4
Hysterectomy / oophorectomy	1	0.3	0	0.0
Premenstrual hunger	1	0.3	0	0.0
Progesterone replacement	1	0.3	0	0.0
Appetite / Hunger	0	0.0	1	1.4

Excluding women missing percentage weight change (*n* = 32), women with percentage weight gain ≤5% (*n* = 12), women not giving reasons for weight gain (*n* = 72), women with unknown parity status (*n* = 5). Women could have been excluded for than one of these reasons. Participants could give multiple reasons for weight gain

^a^Includes all women but only *n* = 152 women known to be peri/postmenopausal women. Of peri/postmenopausal women, 58/152 (38.2%) gave menopause/HRT as a reason for their weight gain

^b^Includes all women but only *n* = 36 women known to be peri/postmenopausal women. Of peri/postmenopausal women, 15/36 (41.7%) gave menopause/HRT as a reason for their weight gain

For the sub-cohort who were peri/postmenopausal and who provided reasons for weight gain (*n* = 188), the proportion stating menopause / HRT as a reason for weight gain was 38.8% (Table 5). Peri/postmenopausal women were less likely to report children / childcare / pregnancy, work / study, and stress as reasons for their weight gain. For premenopausal women (*n* = 211), Menopause / HRT as a reason for weight gain had been replaced in the main ten reasons again by takeaways / ready meals.

**Table 5:** Self-reported reasons for weight gain: peri/post- and premenopausal women

Reason	Peri/postmenopausal women (***n*** = 188)		Premenopausal women (***n*** = 211)	
	Number stating reason	% stating reason	Number stating reason	% stating reason
Children / Childcare / Pregnancy	93	49.5^a^	131	62.1^b^
Inactivity	84	44.7	82	38.9
Menopause / HRT	73	38.8	–	–
Comfort or boredom eating	64	34.0	89	42.2
Portion Size	60	31.9	70	33.2
Stress	44	23.4	66	31.3
Marriage / settling down	42	22.3	54	25.6
Work / Study	30	16.0	51	24.2
Bereavement	28	14.9	45	21.3
Depression	28	14.9	36	17.1
Alcohol	28	14.9	20	9.5
Takeaways / Ready meals	25	13.3	36	17.1
Lack of time to prepare food	21	11.2	34	16.1
Health problems / Medications	20	10.6	18	8.5
Giving up smoking	18	9.6	23	10.9
Food Preferences (own/family)	17	9.0	26	12.3
Cooking skills	4	2.1	7	3.3
Eating out	4	2.1	2	0.9
Cost of healthy food	3	1.6	7	3.3
Lack of sleep / tiredness	3	1.6	3	1.4
Carer	3	1.6	1	0.5
Social life / holidays	3	1.6	1	0.5
Contraceptives	2	1.1	15	7.1
Sweet tooth	2	1.1	1	0.5
Fertility treatment	1	0.5	3	1.4
Appetite / Hunger	1	0.5	0	0.0
Divorce	0	0.0	2	0.9
Hysterectomy / oophorectomy	0	0.0	1	0.5
Premenstrual hunger	0	0.0	1	0.5
Progesterone replacement	0	0.0	1	0.5

Excluding women missing percentage weight change (*n* = 32), women with percentage weight gain ≤5% (*n* = 12), women not giving reasons for weight gain (*n* = 72), women with menopausal status unknown (*n* = 6). Women could have been excluded for than one of these reasons. Participants could give multiple reasons for weight gain

^a^Includes all women but only *n* = 152 women known to have children. Of those with children, 93/152 (61.2%) gave Children / Childcare / Pregnancy as a reason for their weight gain

^b^Includes all women but only *n* = 174 women known to have children. Of those with children, 131/174 (75.3%) gave Children / Childcare / Pregnancy as a reason for their weight gain

Comparison of weight gain reasons between women with moderate (lifetime risk of breast cancer 17–30% and) versus high risk (> 30% lifetime risk) of breast cancer showed that the proportions of women at moderate risk stating many of the reasons were higher than amongst women at high risk of breast cancer (for example comfort or boredom eating 43.0 vs. 34.2%, portion size 37.0 vs. 29.0%) (Table 6). The main ten reasons for both groups differed only by the tenth reason which was depression for women at moderate risk, and takeaways / ready meals for women at high risk of breast cancer.

**Table 6:** Self-reported reasons for weight gain: moderate and high risk of breast cancer

Reason	Moderate risk (17–30% lifetime risk) (***n*** = 200)		High risk (> 30% lifetime risk) (***n*** = 193)	
	Number stating reason	% stating reason	Number stating reason	% stating reason
Children / Childcare / Pregnancy	115	57.5^a^	105	54.4^b^
Inactivity	83	42.5	83	41.5
Comfort or boredom eating	86	43.0	66	34.2
Portion Size	74	37.0	56	29.0
Stress	66	33.0	44	22.8
Work / Study	50	25.0	31	16.1
Marriage / settling down	46	23.0	48	24.9
Bereavement	45	22.5	28	14.5
Menopause / HRT	40	20.0^c^	30	15.5^d^
Depression	38	19.0	26	13.5
Lack of time to prepare food	35	17.5	20	10.4
Takeaways / Ready meals	34	17.0	27	14.0
Alcohol	30	15.0	18	9.3
Food Preferences (own/family)	30	15.0	13	6.7
Health problems / Medications	22	11.0	16	8.3
Giving up smoking	17	8.5	24	12.4
Cooking skills	7	3.5	4	2.1
Cost of healthy food	7	3.5	3	1.6
Contraceptives	7	3.5	10	5.2
Eating out	4	2.0	2	1.0
Lack of sleep / tiredness	3	1.5	3	1.6
Carer	3	1.5	1	0.5
Fertility treatment	3	1.5	1	0.5
Social life / holidays	2	1.0	2	1.0
Sweet tooth	2	1.0	1	0.5
Appetite / Hunger	1	0.5	0	0.0
Divorce	1	0.5	1	0.5
Hysterectomy / oophorectomy	1	0.5	0	0.0
Premenstrual hunger	0	0.0	1	0.5
Progesterone replacement	0	0.0	1	0.5

Excluding women missing percentage weight change (*n* = 32), women with percentage weight gain ≤5% (*n* = 12), women not giving reasons for weight gain (*n* = 72), women with unknown risk (*n* = 13). Women could have been excluded for than one of these reasons. Participants could give multiple reasons for weight gain

^a^Includes all women but only *n* = 168 women known to have children. Of those with children, 115/168 (68.5%) gave Children / Childcare / Pregnancy as a reason for their weight gain

^b^Includes all women but only *n* = 153 women known to have children. Of those with children, 105/153 (68.2%) gave Children / Childcare / Pregnancy as a reason for their weight gain

^c^Includes all women but only *n* = 86 women known to be peri/postmenopausal women. Of peri/postmenopausal women, 40/86 (46.5%) gave menopause/HRT as a reason for their weight gain

^d^Includes all women but only *n* = 98 women known to be peri/postmenopausal women. Of peri/postmenopausal women, 30/98 (30.6%) gave menopause/HRT as a reason for their weight gain

## Discussion

In this analysis of women at increased risk of breast cancer who had joined weight loss studies in Manchester, most women gave multiple perceived reasons for their weight gain. The main causes of weight gain as perceived by the study participants were related to pregnancy and childcare, a lack of PA, and comfort and boredom eating. The predominant reasons for weight gain for sub-cohorts with/without children, pre/peri or postmenopausal and moderate/high risk of breast cancer were broadly similar.

Our recent review of weight gain in 18–35 year old women highlighted many of the reasons also reported in the current analysis [8]. Pregnancy and motherhood are amongst the life events associated with the largest amount of weight gain, with women in the UK weighing a mean of 3.5 (SD 6.2) kg heavier than their pre-pregnancy weight at 6 months postpartum (*n* = 12,583) [27]. Motherhood is associated with a decrease in levels of PA [28, 29], mainly due to lack of time [30–32], which could contribute to further weight gain after pregnancy. This aligns with the current findings as children / childcare / pregnancy was the major reason given by women for their weight gain in both the whole cohort (56%) and the sub-cohort with children (68%). Inactivity was the next most cited reason by 43% of the whole cohort. Figures for England state that 34% of adult females (age 19–84) do not meet government guidelines for physical activity [33] (> 150 min of moderate or 75 min of vigorous intensity activity per week, or a combination of both) and a summary of evidence by the World Cancer Research Fund showed that there was convincing evidence that walking protects against weight gain and probable evidence for aerobic PA [34].

Thirty-two percent of women gave portion size as a reason for their weight gain. The link between portion size and weight gain is complex due the effects of other factors such as energy density and frequency of consumption, but reviews of the literature suggest that there is enough evidence to implicate portion size as an important component in the development of overweight and obesity [35, 36].

Finding effective ways to support women avoid weight gain during adulthood is a priority for women identified at increased risk of breast cancer. Our recent review summarised previous research into behavioural weight gain prevention interventions but none are targeted to this population [8]. The present results indicate that weight gain prevention programmes for this population should cover the same issues as those targeting the general population. Interventions based around pregnancy and postpartum have the strongest evidence base and a number of interventions could progress to practice. Evidence for interventions targeting other life events or general weight gain in young women is weaker and hampered by poor quality studies [8]. In addition to a standard behavioural approach to prevent weight gain, other emerging weight management methods such as third-wave cognitive behaviour therapies [37] and pharmacotherapy, for example GLP-1 receptor agonists in reduced doses [38], may also be effective in the field of weight gain prevention.

### Strengths

The strength of the current analysis is that combining data from a number of studies increased the sample size to over 500 women, with the majority (85%) providing reasons for weight gain. This is the first overview of reasons for weight gain specific to the population of women at increased risk of breast cancer.

### Limitations

There is likely to be an overlap between many of the reasons provided, i.e. inactivity is often attributed to lack of time which is commonly associated with motherhood and childcare [30–32]. Lower PA is also associated with marriage and cohabiting in women [39, 40]. Comfort or boredom eating (mentioned by 38% of the cohort) is often associated with stress (27%) and depression (16%) [41].

The generalisability of the findings to high-risk populations is limited for a number of reasons. Women joined the included studies because they were interested in losing weight by joining dietitian-led weight loss programmes. The majority of participants were white and the population was skewed towards the least deprived quintile, as is a recognised problem in health research [42].

Weight at study entry was reliably measured as per study protocols on calibrated scales, however weight in young adulthood was recalled therefore could be inaccurate, although studies have shown recalled weights to be fairly accurate on a population level [43].

Providing tick box options could have been leading and altered results, for example attending university is often associated with weight gain in women [44, 45] but was not included in the tick boxes which may explain why it was not mentioned by any of the study participants. Other known mediators of weight gain which were not in the questionnaire include lack of knowledge and skills around food and cooking, anxiety, and neural responses [8]. Primacy bias, where respondents are more likely to choose the first few response options when presented with a list, could also have affected the present results. Women could select multiple reasons for weight gain with no weighting given therefore all reported reasons were given equal weight and analysis was performed on frequency. This may have resulted in a loss of detail regarding the relative importance of each reason but it may be unfeasible to ask women to retrospectively recall the amount of weight gain linked with the different reasons. Some of the sub-cohorts we analysed were small, for example there were only 76 women with no children.

We were unable to compare reasons between high risk and population risk women. Only two of the six trials also included population-risk women so as there was not a large enough pool of these women. A stronger study design would have included adequate numbers of women in both groups to allow statistical comparison but it is interesting that the current results show similar findings to the literature about the general female population.

## Conclusions

Limiting weight gain that is frequently experienced by young women could reduce the burden of breast cancer and other diseases. Our analysis has highlighted common reasons women at increased risk of breast cancer attribute to their gain weight. This will help inform the design of future interventions to support women to avoid weight gain. Future research should focus on developing and testing such interventions thus reducing weight gain particularly in women identified at increased risk of breast cancer which will lead to a reduction in the burden of breast cancer.

## Data Availability

The datasets analysed for this article are available in the Figshare repository (10.48420/19614531.v1), apart from the Lifestyle Study for which the dataset is not publicly available as participants on this study did not consent to anonymous data sharing.
